# Multiple Papeln an den Oberschenkeln eines Erwachsenen

**DOI:** 10.1007/s00105-026-05728-9

**Published:** 2026-06-19

**Authors:** Christiane Cussigh, Alexander Enk, Ferdinand Toberer

**Affiliations:** Klinik für Dermatologie, Venerologie und Allergologie, Universitätsklinikum Heidelberg (UKH), Ruprecht-Karls-Universität, Im Neuenheimer Feld 440, 69120 Heidelberg, Deutschland

**Keywords:** Adulte Xanthogranulome, Multiple Xanthogranulome, Multiple Papeln, Gelbliche Papeln, Nicht-Langerhans-Zell-Histiozytose, Adult xanthogranuloma, Multiple xanthogranulomas, Multiple papules, Yellowish papules, Non-Langerhans cell histiocytosis

## Abstract

Das erstmalige Auftreten eruptiver Xanthogranulome im Erwachsenenalter ist seltener als im Kindesalter. Die Pathogenese ist noch ungeklärt. Einzelne Fallberichte beschreiben Assoziationen zu hämatologischen Erkrankungen. Die Histologie weist neben einem dermalen histiozytären Entzündungsinfiltrat auch mehrkernige Touton-Riesenzellen auf. Nachfolgend wird über einen Fall von multiplen, eruptiven adulten Xanthogranulomen mit partieller Spontanregression berichtet.

## Abstract (en)

Initial presentation of eruptive xanthogranulomas in adulthood is less common than in childhood. The pathogenesis is not yet fully understood. Isolated case reports with associations to hematological disorders have been described. Histology reveals a dermal histiocytic inflammatory infiltrate as well as multinucleated Touton giant cells. We report a case of multiple eruptive xanthogranulomas in an adult with partial spontaneous regression.

## Anamnese

**Abb. 1:**
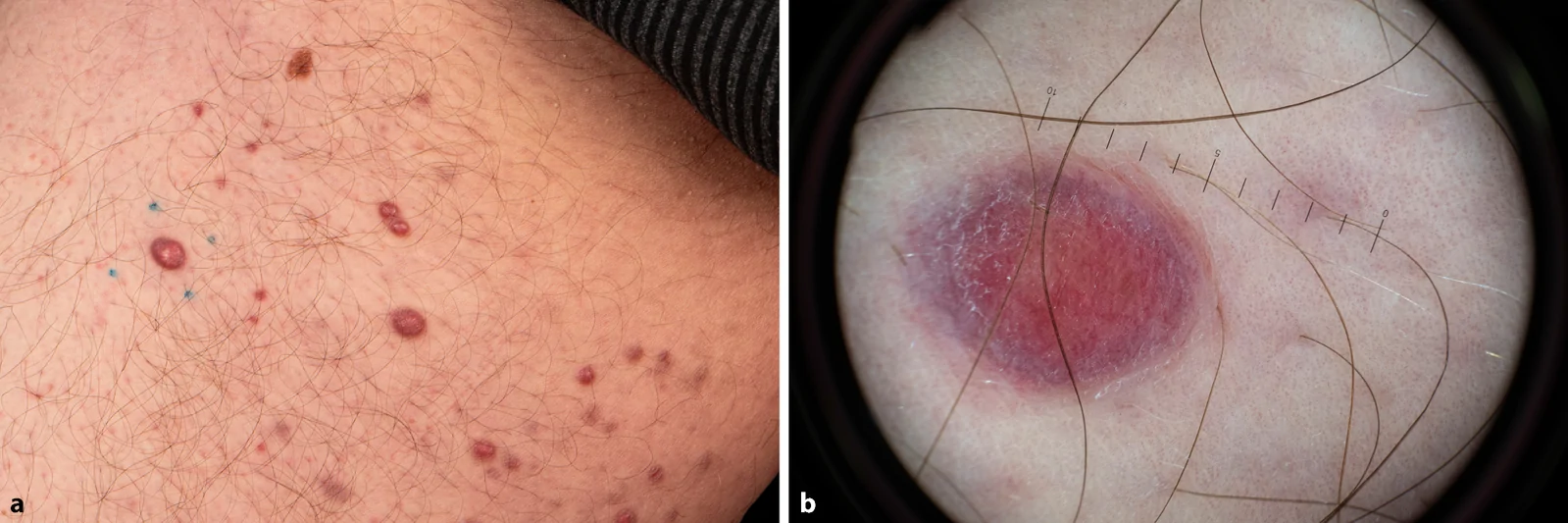
Klinik: Es zeigen sich multiple flache und kugelige livide sowie teilweise gelbliche Papeln an der Innenseite des rechten Oberschenkels (**a**). Auflichtmikroskopisches Bild einer lividen Papel mit gelblichem Zentrum (**b**)

Ein 51-jähriger Patient wurde ambulant vorstellig aufgrund von seit 6 Monaten bestehenden, neu aufgetretenen gruppierten Papeln an beiden Oberschenkelinnenseiten. Diese seien spontan entstanden und würden weder Schmerzen noch Juckreiz verursachen (Abb. 1a). Einzelne Papeln würden sich spontan zurückbilden. Internistische Vorerkrankungen wurden verneint.

## Untersuchung

Bei der Inspektion der unteren Extremitäten zeigten sich multiple gruppierte Papeln an beiden Oberschenkelinnenseiten (rechts mehr als links), die in der Auflichtmikroskopie teils eine gelb-erythematöse und teils eine livide Farbe aufwiesen (Abb. 1b). Der Durchmesser der Knötchen erstreckte sich von 2 mm bis ca. 8 mm.

## Diagnostik

**Abb. 2:**
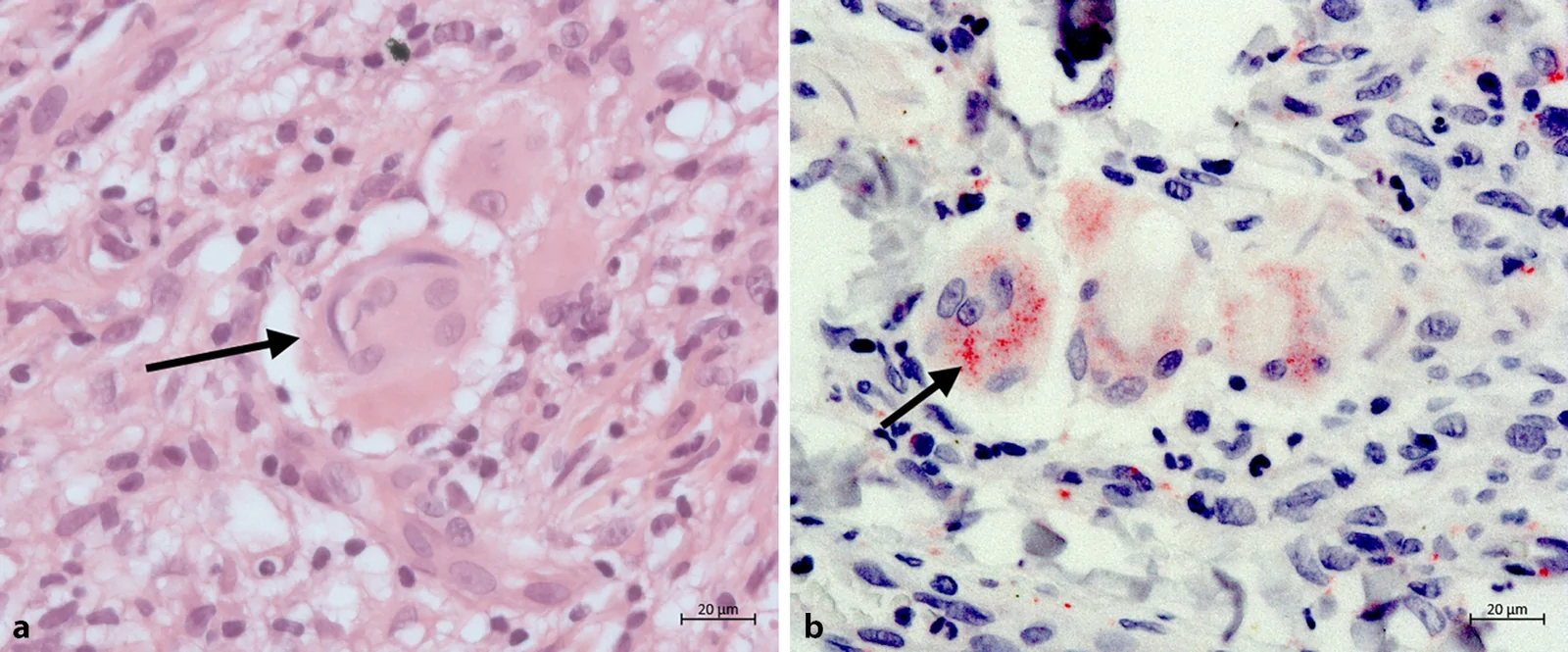
Histologischer Befund: Die Histologie zeigt einen dermalen Tumor aus histiozytären Zellen, die teils epitheloidzellig und teils plump spindelzellig sind (**a**, HE, Vergr. 400:1). Der *Pfeil* (→) zeigt auf eine Riesenzelle. Die Adipophilin-Färbung  (**b**, Vergr. 400:1) zeigt sich deutlich positiv (→).

Bei Verdacht auf eruptive adulte Xanthogranulome erfolgte eine laborchemische Nüchternbestimmung folgender Werte: Blutfette (Triglyceride, Gesamtcholesterin, HDL und LDL), TSH, HbA_1c_, Diff-BB. Hier zeigten sich keine für die Hautveränderungen ursächlichen Auffälligkeiten. Des Weiteren wurde eine etwas länger bestehende Papel am rechten Oberschenkel medial in Lokalanästhesie zur histopathologischen Untersuchung exzidiert. Die Histologie (Abb. 2a) zeigt einen knotigen dermalen Tumor, bestehend aus leicht epitheloiden und teils spindelzelligen histiozytären Zellen sowie mehrkernigen Riesenzellen (Abb. 2a). Letztere sind positiv für Adipophilin (Abb. 2b). Immunhistochemisch waren die Zellen überwiegend positiv für CD68. In Zusammenschau von Anamnese und Diagnostik wurde die Diagnose eruptiver adulter Xanthogranulome gestellt

## Therapie und Verlauf

Aufgrund anamnestisch berichteter Tendenz zur spontanen Regression der Xanthogranulome wurde mit dem Patienten ein abwartendes Verhalten besprochen. Drei Monate nach Erstvorstellung war weiterhin eine Regressionstendenz zu verzeichnen.

## Diskussion

Eruptive Xanthogranulome beim Erwachsenen stellen eine seltene Variante der Nicht-Langerhans-Zell-Histiozytosen dar und sind deutlich seltener als die klassische juvenile Form [6, 9]. Diese manifestiert sich meist als solitäre oder multiple Papeln oder Knoten insbesondere an Rumpf, Kopf und Nacken [6, 9]. Bei Erwachsenen werden multiple Läsionen seltener gesehen als solitäre [5, 7]. Im vorliegenden Fall handelt es sich um multiple Läsionen an der unteren Extremität. Während bei Kindern zusätzlich extrakutane Manifestationen der Xanthogranulome beschrieben werden, scheinen adulte Xanthogranulome nicht mit extrakutaner Beteiligung einherzugehen [2, 9]. Bei eruptiven Papeln kommen differenzialdiagnostisch auch Mollusken, eruptive Keratoakanthome, eruptive Xanthome, eine kutane Sarkoidose, Dermatofibrome sowie eine Leucaemia cutis in Betracht.

Histopathologisch zeigen Xanthogranulome ein dichtes dermales Infiltrat aus Histiozyten, häufig begleitet von Touton-Riesenzellen und einem gemischten Entzündungsinfiltrat [4, 8]. Immunhistochemisch sind die Zellen typischerweise CD68-positiv und CD1a- und S100-negativ, was eine Abgrenzung zur Langerhans-Zell-Histiozytose ermöglicht [4]. Die Pathogenese eruptiver Xanthogranulome beim Erwachsenen ist bislang nicht vollständig geklärt [1]. Es wird angenommen, dass die Xanthogranulome einen reaktiven Prozess auf unbekannte Stimuli darstellen [5]. In Fallberichten ist eine Assoziation von Xanthogranulomen mit hämatologischen Erkrankungen beschrieben [3, 5, 8, 9]. Shoo et al. vermuten als Ursache hierfür eine Induktion von Zytokinen [8]. Larson et al. erachten erhöhte Gammaglobuline als mögliche Ursache einer Histiozytenproliferation [7]. Da das Auftreten von Xanthogranulomen vor, während oder auch nach dem Erscheinen von hämatologischen Erkrankungen erfolgen kann [8], sollten eine entsprechende hämatologische Abklärung und ggf. auch eine Verlaufskontrolle erfolgen. Der molekulare Pathomechanismus bleibt bis heute unvollständig geklärt. Dies bietet Grundlage für zukünftige wissenschaftliche Untersuchungen.

Während in einzelnen Fallberichten beschrieben wird, dass sich adulte Xanthogranulome nicht spontan zurückbilden [1, 5], ist im vorliegenden Fall eine spontane Regression zu verzeichnen. Für eruptive Xanthogranulome besteht keine zwingende Behandlungsindikation. Es gibt Fallberichte über eine erfolgreiche orale Therapie mit 20 mg Isotretinoin 1‑mal täglich mit Besserung nach nur 2 Monaten [1]. In Einzelfällen sind zudem auch Laserbehandlungen oder chirurgische Exzisionen bei kosmetischer Beeinträchtigung oder ausgedehnten Befunden erfolgt [2]. Hierfür existieren allerdings keine standardisierten Therapieempfehlungen.

## Fazit für die Praxis

- Eruptive Xanthogranulome des Erwachsenen stellen eine seltene, aber wichtige Differenzialdiagnose multipler, eruptiver Papeln dar.
- Die Diagnose des eruptiven Xanthogranuloms des Erwachsenen basiert auf der Kombination aus klinischem Erscheinungsbild und histopathologischen Befunden.
- Aufgrund möglicher Assoziationen mit hämatologischen Erkrankungen, wenn auch selten beschrieben, sollte eine diesbezügliche Abklärung erfolgen.

## Data Availability

Alle dieser Arbeit zugrunde liegenden Daten sind in diesem Artikel enthalten.
